# Health literacy in patients with epilepsy: a narrative review of current status, influencing factors, and future directions

**DOI:** 10.3389/fneur.2026.1885747

**Published:** 2026-06-29

**Authors:** Nan Wen, Rui Wang, Yueyue He, Qian Jiang, Jianyu Peng, Ling Feng

**Affiliations:** 1 Department of Neurology, West China Hospital, Sichuan University, Chengdu, Sichuan, China; 2 West China School of Nursing, Sichuan University, Chengdu, Sichuan, China; 3 Department of Emergency Medicine, West China Tianfu Hospital, Sichuan University, Chengdu, Sichuan, China; 4 Department of Neurology, West China Tianfu Hospital, Sichuan University, Chengdu, Sichuan, China

**Keywords:** epilepsy, health literacy, influencing factors, intervention strategies, narrative review, self-management

## Abstract

Epilepsy is a common chronic neurological disorder that imposes a significant burden on patients’ physical and mental health, and low health literacy is closely associated with poor medication adherence, increased seizure frequency, and reduced quality of life in patients with epilepsy. Despite the growing number of studies on health literacy among patients with epilepsy, there is a lack of a comprehensive narrative summary that systematically integrates the factors influencing health literacy and corresponding intervention strategies, hindering the formulation of targeted health management measures. This narrative review aims to summarize current research on health literacy among patients with epilepsy, clarify the key influencing factors, and systematically sort out the common intervention strategies. We searched databases including PubMed, Embase, and Web of Science, screening Chinese and English studies published from the inception of the databases through March 31, 2026, and included studies focusing on epilepsy patients’ health literacy levels, influencing factors, and intervention effects. The results show that individual characteristics (for example, age, education level, cognitive function), family and social support, and healthcare resource accessibility are the main factors affecting health literacy in epilepsy patients, and comprehensive intervention measures can effectively improve patients’ health literacy and self-management ability. This review also highlights the limitations of existing studies, such as the lack of standardized assessment tools and long-term follow-up data, and proposes feasible directions for future research. In conclusion, this narrative review provides a comprehensive reference for clinical medical staff to formulate targeted health literacy intervention strategies for patients with epilepsy and promotes the standardized development of epilepsy health management.

## 1 Introduction

Epilepsy is one of the most common chronic neurological disorders. The global prevalence of epilepsy has reached 50 million people, with nearly 80% of cases occurring in low- and middle-income countries (1), and nearly two-thirds of patients do not receive standardized diagnosis and treatment (2). It is estimated that people with epilepsy face a risk of premature death up to three times higher than the general population (3), and approximately half of adult patients with epilepsy have at least one other physical or mental health condition, such as anxiety or depression (4, 5). In addition, people with epilepsy also face social stigma (6) and unequal access to healthcare resources (7). Long-term, standardized self-management remains a core component of epilepsy control (8).

Health literacy is a key determinant of long-term outcomes in epilepsy. Patients with higher health literacy better understand their condition, adhere to treatment plans, and cope with various challenges (9). Multiple studies have consistently shown that low health literacy is associated with adverse outcomes in epilepsy (10), including reduced quality of life (11), increased stigma (12), and a higher risk of hospitalization (13). Conversely, higher health literacy is associated with lower rates of missed medication doses and fewer seizures (14). Therefore, improving health literacy among people with epilepsy can lead to better health outcomes (15).

Despite growing recognition of the importance of health literacy in epilepsy care, current research still has limitations. Most studies adopt unidimensional approaches, focusing solely on the level of health literacy or its association with a single outcome measure, such as medication adherence (8, 16, 17), rather than treating health literacy as a multidimensional concept encompassing functional, interactive, and critical dimensions. Consequently, significant conceptual and methodological variations exist across studies. Moreover, digital health literacy has not yet received sufficient attention, resulting in a significant disconnect between rapidly evolving digital health interventions and patients’ actual ability to use these tools.

Given these limitations, a comprehensive synthesis of the current evidence is needed. This narrative review aims to summarize the current status, influencing factors, and intervention strategies for health literacy in people with epilepsy, thereby providing a reference for identifying high-risk populations and guiding future research and practice.

## 2 Materials and methods

**Figure 1:**
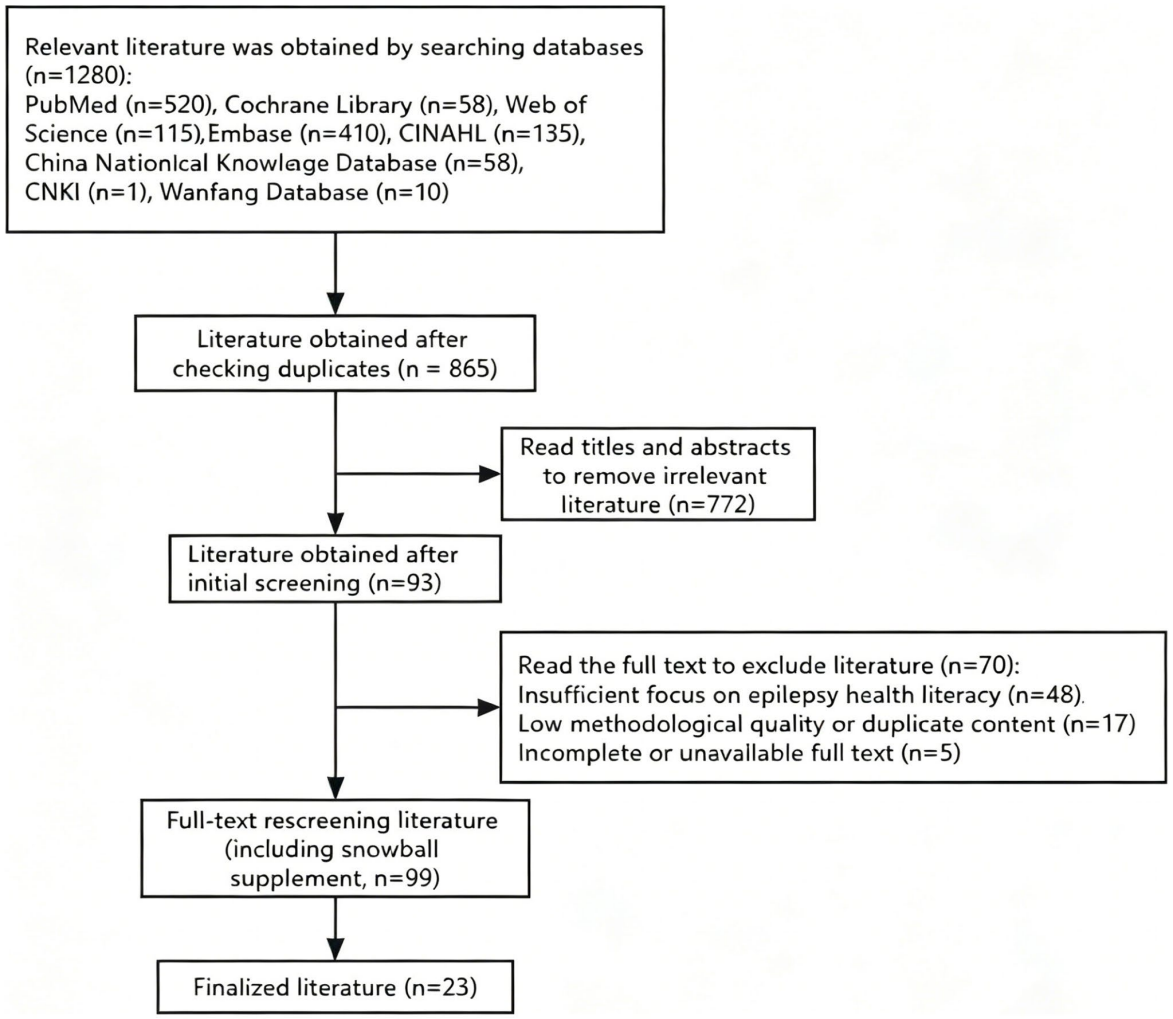
Flowchart of the literature selection process.

This study adopted a narrative review approach, which is well-suited for synthesizing qualitative and heterogeneous evidence. This approach offers greater flexibility in examining diverse intervention strategies while accounting for the dynamic and evolving nature of the evidence base (18). The field of epilepsy health literacy is characterized by inconsistent definitional frameworks, a variety of assessment tools, and substantial clinical and methodological heterogeneity across study designs (cross-sectional, qualitative, and quasi-experimental). Moreover, our primary objective is to provide a comprehensive overview of definitions, influencing factors, and intervention strategies, with a focus on identifying conceptual gaps and emerging trends, a goal that a narrative review is better positioned to achieve. The detailed literature screening process is presented in Figure 1.

A comprehensive literature search was conducted in PubMed, the Cochrane Library, Web of Science, Embase, CINAHL, China National Knowledge Infrastructure (CNKI), Wanfang Database, and the China Biomedical Literature Database (CBM) from database inception to March 31, 2026. The search strategy used the following English keywords: “epilepsy,” “health literacy,” “eHealth literacy,” “self-management,” “medication adherence,” “influencing factors,” and “intervention strategies.” Studies were included if they were original research, systematic reviews, meta-analyses, and clinical guidelines focusing on health literacy among patients with epilepsy; peer-reviewed articles published in English or Chinese; and high-quality, highly cited, and representative studies in this field. Exclusion criteria comprised conference abstracts, case reports, letters, commentaries, editorials, non-peer-reviewed gray literature, articles not related to epilepsy health literacy, and literature not published in English or Chinese. Literature screening was performed using a two-stage preliminary review process combined with snowball sampling of key references.

## 3 Concept of health literacy among patients with epilepsy

Since the concept of health literacy (HL) was first proposed by the American scholar Simonds (19), its definition has evolved considerably. The Institute of Medicine (IOM) defines HL as the ability of individuals to obtain, process, and understand basic health information and services needed to make appropriate health decisions (20). The World Health Organization (WHO) emphasizes that HL represents cognitive and social skills that determine an individual’s motivation and capacity to access, understand, and use information to promote and maintain health (21). Importantly, HL is not merely a reflection of individual skills; it also depends heavily on the accessibility of the healthcare system, the communication skills of healthcare professionals, and the complexity of health information (22).

Nutbeam’s hierarchical model categorizes HL into three levels—functional, interactive, and critical-revealing how patients progress from passive compliance to active decision-making (23). This model has become a key theoretical framework for HL research and is widely applied in chronic disease studies (24, 25). However, the full application of Nutbeam’s multidimensional framework to epilepsy is constrained by the available evidence, which overwhelmingly focuses on functional health literacy. Interactive and critical health literacy remain severely understudied. Therefore, this review is weighted toward functional health literacy, and we identify the absence of interactive/critical data as a key gap.

In the context of rapidly advancing digital health technologies, the concept of HL has further expanded. Specifically, e-health literacy, the ability to search for, evaluate, and apply health information through digital channels, has become an indispensable core competency in modern healthcare (15). It enables patients to use telemedicine platforms, participate in remote EEG monitoring and virtual consultations, and access specialized neurology services without geographical or time constraints, thereby breaking down barriers caused by the uneven distribution of medical resources (26, 27). This is particularly important for patients in rural and medically underserved areas. In addition, digital tools provide automated medication reminders, personalised identification of seizure triggers, and adaptive lifestyle guidance, supporting precise and individualised self-management (28).

Although the multidimensional nature of HL is increasingly recognised, its application in epilepsy requires special consideration. Unlike common chronic diseases such as hypertension or diabetes, epilepsy-as a chronic neurological disorder-is characterized by unpredictable seizures, the need for lifelong treatment, significant social stigma, and potential cognitive and psychological comorbidities (29, 30). These factors collectively shape the unique implications of HL in epilepsy. Therefore, how individuals with epilepsy acquire, understand, and apply health information is of great importance for improving patient outcomes and reducing disease burden.

## 4 Current status and assessment tools of health literacy among people with epilepsy

### 4.1 Current status

Current evidence suggests that HL among people is unevenly distributed across different functional dimensions. Regarding disease perception, patients’ understanding of the nature of epilepsy is strongly influenced by regional culture and access to healthcare. A large-scale multicenter study conducted in China further confirmed that the overall prevalence of low health literacy was 33.6%, with a rate of 28% among people with epilepsy (31). A large-scale survey across ten European countries found that although patients’ overall disease knowledge was generally adequate, gaps persisted on core issues such as medication and etiology (23). In Ethiopia, 47.37% demonstrated good understanding of epilepsy, yet only 46.83% held a positive attitude toward the condition, indicating a substantial knowledge–attitude gap (32). In contrast, a study in Palestine reported that 52.1% of respondents possessed good knowledge of epilepsy, and 82.5% held positive attitudes toward people with epilepsy (33).

With regard to medication management, although most patients recognized the importance of medication, approximately 25% of cases with poor seizure control are associated with poor adherence rather than medication ineffectiveness (34). Among epilepsy patients in six rural provinces and municipalities in China, 74.17% demonstrated good medication adherence (35). Seizure first-aid management is also concerning. A cross-sectional study in China indicated that only 29.6% of patients could correctly perform first-aid procedures, and only 21.7% considered themselves prepared for emergencies (9).

Among special populations, elderly patients with epilepsy generally have lower HL than younger patients. This difference is associated with comorbidities, cognitive decline, and low acceptance of new technologies (36). Women of childbearing age with epilepsy harbor many misconceptions regarding the teratogenic risks of medications and the safety of breastfeeding (37). Additionally, Toker et al. (38) also found that 40.2% of parents of children with epilepsy had insufficient health literacy.

Importantly, although more than 90% of people with epilepsy wish to obtain information about their condition, the majority report not receiving the information they need regarding non-pharmacological treatments and psychosocial issues (39). This contradiction between patients’ insufficient health literacy and their strong desire to obtain disease-related information should be a core focus for clinicians. Finally, current studies on health literacy in epilepsy predominantly focus on functional health literacy, which involves the acquisition and comprehension of disease-related knowledge, as shown in Table 1. Studies on interactive and critical health literacy remain limited, and this gap also deserves close attention from healthcare provider.

**Table 1:** Summary of health literacy domains, tools and assessment outcomes.

Health literacy domain	Definition in epilepsy context	Example measurement tools	Example outcomes assessed	Notes/Limitations
Functional health literacy	Basic reading, writing, and numeracy skills to understand epilepsy-related information (e.g., medication labels, seizure action plans, appointment slips).	REALM/REALM-R TOFHLA HLS-EU-Q (functional subscale) HLS (general) EKP-G (epilepsy-specific knowledge)	Correct interpretation of prescription labels; understanding of seizure first-aid instructions; basic knowledge of epilepsy causes and triggers; ability to follow written discharge instructions	Most widely used tools (REALM, TOFHLA) focus on reading and numeracy. EKP-G measures declarative knowledge only (55 true/false items) and is not a true health literacy instrument; it captures only a portion of functional HL. No comprehensive, globally validated epilepsy-specific HL tool exists (15, 44).
Interactive health literacy	Advanced cognitive and social skills to actively extract information from diverse sources (e.g., asking clinicians questions, seeking information from multiple channels) and apply it to changing circumstances.	HLS-EU-Q (interactive subscale) HLQ (e.g., “actively managing my health”) eHEALS (information seeking component)	Asking relevant questions during medical consultations; seeking second opinions or additional information; adapting self-management to life changes (pregnancy, employment, driving) ; Using online patient portals	No validated epilepsy-specific interactive HL tool. General scales have been used in epilepsy but lack epilepsy-specific content validation.
Critical health literacy	Ability to critically analyse health information, recognise social and structural determinants of health (e.g., stigma, healthcare system barriers), and advocate for personal or collective change.	No validated epilepsy-specific instrument Qualitative interviews on stigma and advocacy	Recognising stigmatising attitudes in society Challenging misconceptions about epilepsy Advocating for workplace/school accommodations Participating in epilepsy advocacy or support groups	Critical HL remains almost entirely unexamined in epilepsy research. No quantitative tool has been validated for this population.
Digital/eHealth literacy	Ability to seek, find, understand, and appraise health information from electronic sources and to apply that knowledge to address or solve a health problem.	eHEALS	Using telemedicine platforms Evaluating reliability of online epilepsy information Using seizure diary apps Participating in online patient communities	eHEALS is self-reported and measures perceived skills, not objective performance. Few epilepsy-specific digital literacy studies exist.

### 4.2 Assessment tools

To date, no comprehensive, globally validated health literacy assessment tool has been developed specifically for people with epilepsy (15). Given that epilepsy is a chronic condition, most existing studies have used general-purpose scales, including the Rapid Evaluation of Adult Medical Literacy (REALM) (40), the Test of Functional Health Literacy for Adults (TOFHLA) (11), the Health Literacy Survey (HLS-EU-Q) (10, 41), the Health Literacy Scale (HLS) (42), and the eHealth Literacy Scale (eHEALS) (43). While these scales have been applied to epilepsy patient populations, they primarily assess recognition of medical terminology and basic reading skills. They lack evaluation of interactive and critical health literacy, as well as content specific to epilepsy patients.

Knowledge about epilepsy is generally considered part of functional health literacy. However, few epilepsy-specific assessment tools are available. The most widely used is the Epilepsy Knowledge Profile—General Version (EKP-G), developed by Jarvie et al. (44). This scale consists of 55 true/false questions covering both medical and social aspects of epilepsy, with a Cronbach’s alpha of 0.82 and a test–retest reliability of 0.87. Although the EKP-G has become the gold standard for assessing basic knowledge of epilepsy due to its high content specificity, it is not a true health literacy assessment tool. It measures only a portion of functional health literacy and completely lacks assessment of interactive, critical, and e-health literacy.

## 5 Factors influencing health literacy among people with epilepsy

Health literacy is a complex outcome resulting from the interaction between individual characteristics, family and social environments, and the healthcare system, as shown in Table 2. Identifying these factors is a prerequisite for developing targeted intervention strategies and improving the health literacy of people with epilepsy.

**Table 2:** Summary of factors influencing health literacy in patients with epilepsy.

Category	Influencing factor	Key findings	References
Individual characteristics	Age	Children depend on caregivers; younger parental age linked to lower health literacy. Young adults have higher e-health literacy than older adults. Cognitive decline and limited digital skills correlate with lower literacy in older patients.	(43, 45–48)
	Educational background	Lower education significantly associated with lower health literacy. Education is the strongest predictor, surpassing income and residence.	(31, 40)
	Cognitive function	Epilepsy patients have lower health literacy than general population; lower health literacy linked to poorer memory and slower processing speed. Intellectual disability creates severe barriers.	(49, 50)
	Economic income	Low income associated with lower health literacy and poorer drug knowledge; higher income predicts better access and fewer missed doses.	(10, 11, 14, 31, 40, 51)
	Occupation	Government employees more likely to have good epilepsy knowledge (OR = 3.69). Occupation may influence health literacy indirectly via education/income and access to information.	(31, 52)
	Disease-related factors	Seizure frequency predicts lower knowledge. Poor control may limit learning. Polypharmacy may increase literacy but also cognitive load; causality unclear.	(12, 53–55)
Family and social support	Social support	Poor social support is a structural barrier to self-management. Social support plays an empowering role in health literacy and self-management.	(43, 56, 57)
Healthcare resource accessibility	Doctor-patient communication	Patients recall only ~20% of information after consultations; 40–80% forgotten. Barriers worse for patients with intellectual disabilities.	(50, 58)
	Health education resources	Parents struggle to obtain information; provider capacity gaps hinder health literacy. Urban living predicts higher knowledge. Low and middle-income country face severe constraints (weak systems, AED shortages, sociocultural biases). Telemedicine reduces barriers but equity risks remain.	(27, 43, 59–62)
	Readability of online materials	98.7% of online materials exceed recommended 6th-grade reading level, limiting access and shared decision-making.	(63)
	Reliability of information	62.6% obtain information via social media; higher e-health literacy linked to trust in professional websites. Informal channels may spread misinformation.	(64)

### 5.1 Individual characteristics

#### 5.1.1 Age

Children and adolescents have not yet reached full cognitive maturity, so their health literacy therefore depends largely on their caregivers. Among parents of children with epilepsy, younger parental age is associated with lower health literacy scores, although causality cannot be inferred from this cross-sectional finding (43). Young adult patients generally report higher e-health literacy than older adults (45). A systematic review indicates that age negatively affects digital health literacy, particularly in older adults (46). Cognitive decline and limited digital skills correlate with difficulties in understanding complex medical information and using digital management tools, making older patients more likely to have low health literacy (47). However, some studies indicate that vocabulary-based health literacy abilities may stabilize with age, with cognitive function playing a mediating role (48).

#### 5.1.2 Educational background

Multiple studies have shown a significant association between lower educational attainment and lower health literacy among people with epilepsy (40). Patients with less education are more likely to struggle with health-related tasks, such as reading medication labels, understanding medical instructions, and identifying false medical information online (40). A study of 1,120 patients with chronic neurological diseases found that educational attainment was the strongest predictor of health literacy, with an effect that even surpassed other socioeconomic factors such as income and place of residence (31).

#### 5.1.3 Cognitive function

Cognitive function is a key factor specific to epilepsy. People with epilepsy have significantly lower health literacy than the general healthy population. Furthermore, lower health literacy is independently associated with poorer memory and slower information processing speed, suggesting that cognitive impairment directly affects patients’ ability to access, understand, and apply health information (49). Individuals with intellectual disabilities face even greater barriers to accessing health information (50).

#### 5.1.4 Economic income

Multiple studies confirm that economic income significantly impacts health literacy (10, 31, 40). Low-income patients with epilepsy also exhibit significantly lower knowledge of antiepileptic drugs (51), whereas high-income groups have better access to health information and high-quality medical services (11), in a study of parents of children with epilepsy, Paschal et al. (14) noted that household income was only other predictor associated with reduced missed doses; for every increase of one economic tier in household income, the number of missed doses decreased significantly.

#### 5.1.5 Occupation

Occupation also influences epilepsy-related knowledge. In a community survey, government employees were 3.69 times more likely to possess a good understanding of epilepsy than other occupational groups (52). Furthermore, in univariate analysis, the occupational status of parents of children with epilepsy was significantly associated with health literacy levels, though part of this effect may be mediated by education or income (31). Overall, occupation primarily affects health literacy indirectly through its impact on access to information and healthcare services.

#### 5.1.6 Factors related to disease

Seizure frequency is an independent predictor of patients’ level of epilepsy knowledge and is significantly positively correlated with lower knowledge levels (53). This may be because patients with poor seizure control are too burdened by their condition to devote time to learning about health. However, another study indicates that epilepsy patients with limited health literacy do not necessarily have poorer seizure control, but their quality of life is indeed lower (12). This finding suggests that good seizure control does not necessarily imply a higher level of health literacy, a point worthy of further exploration.

The complexity of drug therapy also influences health literacy. Parents of children taking multiple antiepileptic drugs demonstrate higher medication literacy, possibly because managing complex regimens enhances their information-processing abilities (54). Conversely, polypharmacy may increase cognitive load, which in turn may lower health literacy (55). Future research should explore the causal relationships between disease-related factors (e.g., seizure severity, frequency, and medication types) and health literacy.

### 5.2 Family and social support

Family and social support have been consistently identified as significant factors influencing health literacy in epilepsy research. A qualitative study of 15 parents of children with epilepsy clearly found that information seeking, social support, and effective interaction with healthcare providers as the three core elements for parents to develop adequate health literacy, with social support playing a key role (43). This finding suggests that social support not only serves as an external resource for acquiring health knowledge but also enables caregivers to translate that knowledge into practice.

Furthermore, Perzynski et al. (56) used focus groups to identify structural barriers to epilepsy self-management, explicitly identifying poor social support as one of the core barriers at the community and family levels, and highlighting the link between social integration and positive health behaviors. This finding serves as further evidence of the empowering role that social support plays in health literacy and self-management. Similarly, Sajatovic et al. (57) also noted that social support is a key mechanism for improving self-management of epilepsy, providing empirical support for understanding how social support translates into better health literacy at the operational level.

### 5.3 Healthcare resource accessibility

#### 5.3.1 Communication between doctors and patients

Communication between doctors and patients is a critical factor influencing the health literacy of people with epilepsy. Extensive evidence indicates that doctor-patient communication in the field of epilepsy faces multiple barriers, which profoundly affect the development and application of patients’ health literacy. A study of 50 epilepsy patients who underwent an EEG after their first seizure found that patients could recall only about one-fifth of the information discussed initially during consultations, with 40–80% forgotten afterwards (58). This memory loss is particularly pronounced in the epilepsy population. People with epilepsy and intellectual disabilities face challenges in all four core competencies—accessing, comprehending, evaluating, and applying information—and these difficulties are exacerbated by inadequate communication from healthcare providers and information materials that are not tailored to their needs (50).

#### 5.3.2 Health education resources

The uneven distribution of health education resources limits patients’ access to information and leads to fragmented content. Parents of children with epilepsy often struggle to obtain relevant information and support during the care process, and the lack of capacity among healthcare providers is a major barrier to improving their health literacy (43). Although both patients and caregivers desire more comprehensive epilepsy education, primary and specialized care have failed to meet these needs (59). This problem is particularly pronounced in areas where medical resources are unevenly distributed.

A narrative review on remote follow-up for pediatric epilepsy noted that transportation burdens, caregiver time loss, and limited pediatric neurology services collectively contribute to fragmented disease management, highlighting how unequal access to healthcare resources directly hinders the systematic improvement of health literacy (27). A study in Jordan further demonstrated that living in urban areas significantly predicts higher epilepsy-related knowledge, as urban settings offer broader access to health information sources and social support networks (60). Limited healthcare resources in low- and middle-income countries impose even greater constraints on health literacy (61). In developing countries, low health literacy and knowledge gaps are particularly severe, stemming from weak healthcare systems, shortages of antiepileptic drugs (AEDs) and educational resources, and sociocultural biases (62).

Furthermore, the readability of online patient education materials poses a significant barrier. Seneviratne et al. (63) assessed the readability of 100 online patient education websites regarding epilepsy surgery and found that 98.7% of the sites contained health information with a reading difficulty exceeding the recommended sixth-grade level. This not only limits patients’ independent access to information but also hinders shared decision-making between physicians and patients.

The reliability of health education resources is equally crucial. One study found that 62.6% of epilepsy patients obtain and share health information via social media, while those with higher e-health literacy are more likely to trust professional websites (64). Health knowledge obtained through formal channels, such as hospitals and authoritative medical websites, is more accurate and practical. In contrast, patients who rely on informal channels are prone to being misled by misinformation, which actually hinders the improvement of health literacy. Thus, improving health literacy requires not only ensuring access to information channels but also cultivating patients’ critical thinking skills regarding health information.

## 6 Intervention strategies

### 6.1 Educational interventions

Comprehensive health education currently represents the most fundamental and widely applied intervention approach for enhancing the health literacy and self-management capabilities of epilepsy patients, particularly suitable for special populations with relatively limited information processing abilities. These intervention primarily use face-to-face offline formats (65). Educational materials, such as epilepsy knowledge manuals, simplified drug instruction leaflets, and concise language summaries being widely recognized as essential tools for improving epilepsy-related health literacy (15). A meta-analysis showed that educational interventions significantly improve epilepsy knowledge in children with epilepsy and their parents, and that traditional educational methods are more effective than technology-based approaches like websites and apps when targeting parents (10). In addition, Teach-Back training has been found to be more effective for helping learners master first-aid skills for epilepsy than for imparting theoretical knowledge (66). It is recommended to adopt this method to establish a sound doctor-patient communication environment.

### 6.2 Digital health interventions

Due to their high accessibility, relatively low cost, and ease of scaling, digital health applications have become an important tool for improving health literacy among people with epilepsy. Existing mobile health applications primarily feature two functional modules: an educational module covering epilepsy knowledge and self-management skills, and a behavioral intervention module covering medication reminders, seizure monitoring, and drug side effects. The combination of these two modules can significantly improve patients’ self-management knowledge and medication adherence (16). Online health education yields comparable effects to face-to-face education and is therefore recommended when in-person education is not feasible (67, 68). The Web-Based Epilepsy Education Program (WEEP) has also been shown to be effective in improving knowledge, self-efficacy, attitudes, and e-health literacy among adolescents with epilepsy and their parents (69). Telemedicine, including video and telephone consultations, can safely support medication adjustment, adverse reaction monitoring, epilepsy diary review, consultation services and care coordination (27). They can bridge the gap between patients in resource-limited areas and specialized care and improve access to medical services. One study reduced the treatment gap for epilepsy from 43 to 9% (70). A pharmacist-led smart WeChat group also proved beneficial: patients under 60 years and those taking multiple antiepileptic drugs showed significant gains in disease and medication knowledge, adherence, and seizure-free rates (71).

However, adherence to digital interventions remains a major challenge. A German multicenter randomized controlled trial showed that only 22% of participants in the intervention group completed half of the intervention program, and qualitative feedback indicated that participants preferred personalized content, interactive interfaces, concise text, and stronger reminder functions (72). Another study of 115 epilepsy patients in Germany reported that the most desired features were seizure alerts and data sharing between patients and healthcare providers, although most patients expressed interest in digital health applications, only 37.4% actually used them (73). This phenomenon is possibly related to insufficient digital literacy, lack of operational skills, and privacy concerns. Therefore, digital health interventions should be patient-centered, emphasizing personalisation and interactivity to enhance patients’ willingness to continue using the apps and improve their health literacy.

### 6.3 Current limitations of interventions

As shown in Table 3, current interventions targeting health literacy in epilepsy have several shortcomings. First, most interventions focus on functional health literacy, such as disease awareness and medication knowledge, neglecting the targeted development of multidimensional skills such as doctor-patient communication, health information literacy, and digital health literacy. They also lack a systematic design based on a theoretical framework of health literacy. Second, intervention content often adopts a uniform, standardized model that fails to adequately account for individual differences such as age, educational level, cognitive function, economic status, and cultural background, resulting in a lack of personalized, targeted interventions. Furthermore, digital health interventions are constrained by the digital divide, limiting their application among the elderly, low-income, and less educated populations, while the quality of online medical information varies widely.

**Table 3:** Summary of intervention strategies for improving health literacy in epilepsy.

Intervention type	Specific methods	Key findings/Effects	Limitations/Challenges	References
Educational interventions	Face-to-face education, epilepsy knowledge manuals, simplified drug leaflets, plain language summaries, Teach-Back method	Significantly improves epilepsy knowledge in children and parents. Traditional methods more effective than technology-based approaches for parents. The Teach-Back method is suggested to enhance doctor-patient communication.	Mostly targets functional health literacy; lacks multidimensional design (e.g., communication, digital skills).	(10, 15, 65, 66)
Digital health interventions	Mobile health apps (education + medication reminders, seizure monitoring, side effect tracking), online education programs (WEEP), telemedicine (video/telephone consultations, pharmacist-led smart WeChat groups)	Improves self-management knowledge, adherence, seizure-free rates, e-health literacy, and access. Online education programs effective for adolescents and parents. Telemedicine reduced treatment gap from 43 to 9%. Smart WeChat groups benefit patients <60 years and on polytherapy.	Low adherence (only 22% completed half of one program). Patients desire personalised, interactive content, reminders. Digital divide limits access for elderly, low-income, less educated. Privacy concerns.	(16, 27, 40, 66–70)

## 7 Discussion

This review found that research on health literacy in epilepsy has primarily focused on functional knowledge, whereas the interactive and critical dimensions remain under-explored. A persistent gap exists between knowledge and behavior, particularly regarding emergency seizure response and coping with stigma (9, 34). At the same time, digital health literacy is increasingly emerging as a key determinant, yet access to digital resources and the prevalence of relevant skills vary widely (16, 47).

To address the digital divide, healthcare providers should recognize that the burden of disease may reduce patients’ willingness to adopt health technologies. Therefore, it is crucial to provide personalized e-health literacy support alongside digital tools (74). For patients with low digital literacy, telephone follow-ups can serve as a practical alternative to video consultations (27), although a hybrid model combining both approaches may be more equitable.

The above findings also reveal marked cross-regional variability. Studies from high-income countries more often examine interactive and digital health literacy using validated tools, whereas low and middle-income country studies focus on functional knowledge and report lower literacy levels, strongly associated with education and healthcare access (32, 51, 52). Tools and digital interventions developed in high-income countries may not transfer directly to low and middle-income countries. To overcome such socioeconomic barriers, routine health literacy screening using simple tools is recommended (49), but appropriate assessment tools need to be identified for patients with epilepsy across different economic and cultural contexts. Furthermore, adopting measures such as culturally adapted health education materials for low-literacy populations, task-shifting to community health workers, and utilizing basic mHealth platforms may help prevent the widening of health disparities (15, 26, 61). However, scalability depends on reliable infrastructure, stable funding, cultural adaptation of materials and adequate training programmes (61).

This narrative review has several limitations. First, it did not follow PRISMA guidelines or include a formal quality assessment of the included studies, which may have introduced selection bias. Second, the literature search was restricted to English and Chinese publications; relevant studies in other languages may therefore have been missed. Third, most of the evidence reviewed comes from cross-sectional studies, precluding causal inferences. The lack of longitudinal and interventional studies limits our ability to determine directionality. Fourth, the high reading difficulty of online patient education materials remains a persistent systemic barrier. Finally, the lack of standardized, epilepsy-specific health literacy assessment tools, together with an overemphasis on functional health literacy, further limits the generalizability of the conclusions drawn in this review.

## 8 Conclusion

This narrative review provides a comprehensive synthesis of the current state of research on health literacy among people with epilepsy worldwide. Our analysis confirms that insufficient health literacy is extremely prevalent globally, with particularly notable gaps in medication management, seizure first aid, coping with stigma, and digital health skills. Current assessment tools remain fragmented and lack standardized, disease-specific instruments. Furthermore, health literacy is influenced by the complex interaction of individual, family, healthcare system, and societal factors. However, most existing evidence comes from cross-sectional studies; therefore, causal conclusions cannot be drawn. Future research should prioritise longitudinal designs and intervention trials to determine the direction and mechanisms of these relationships. Although intervention strategies have evolved from simple knowledge dissemination to multimodal, personalized, and digital approaches, high-quality long-term evidence remains scarce. Therefore, this review highlights the urgent need to develop standardised and comprehensive assessment systems, conduct cross-cultural studies, and design targeted interventions-steps that are essential for achieving precise and inclusive epilepsy diagnosis and treatment, as well as improving long-term prognosis for patients worldwide.
